# Manipulating fenestrations in young and old liver sinusoidal endothelial cells

**DOI:** 10.1152/ajpgi.00179.2018

**Published:** 2018-10-04

**Authors:** Nicholas J. Hunt, Glen P. Lockwood, Alessandra Warren, Hong Mao, Peter A. G. McCourt, David G. Le Couteur, Victoria C. Cogger

**Affiliations:** ^1^Centre for Education and Research on Ageing, Concord Repatriation General Hospital, Sydney, Australia; ^2^Biogerontology Group, ANZAC Research Institute, Concord Repatriation General Hospital, Sydney, Australia; ^3^Nutritional Ecology Group, Charles Perkins Centre, University of Sydney, Sydney, Australia; ^4^Department of Medical Biology, University of Tromsø, Tromsø, Norway

**Keywords:** aging, direct stochastic optical reconstruction microscopy, nicotinamide mononucleotide, pharmaceutical, TNF-related apoptosis-inducing ligand

## Abstract

Fenestrations are pores within liver sinusoidal endothelial cells (LSECs) that enable the transfer of substrates (particularly insulin and lipoproteins) between blood and hepatocytes. With increasing age, there are marked reductions in fenestrations, referred to as pseudocapillarization. Currently, fenestrations are thought to be regulated by vascular endothelial growth factor and nitric oxide (NO) pathways promoting remodeling of the actin cytoskeleton and cell membrane lipid rafts. We investigated the effects of drugs that act on these pathways on fenestrations in old (18–24 mo) and young mice (3–4 mo). Isolated LSECs were incubated with either cytochalasin 7-ketocholesterol, sildenafil, amlodipine, simvastatin, 2, 5-dimethoxy-4-iodoamphetamine (DOI), bosentan, TNF-related apoptosis-inducing ligand (TRAIL) or nicotinamide mononucleotide (NMN). LSECs were visualized under scanning electron microscopy to quantify fenestration porosity, diameter, and frequency, as well as direct stochastic optical reconstruction microscopy to examine actin and NO synthase. In young and old LSECs, fenestration porosity, diameter and frequency were increased by 7-ketocholesterol, while porosity and/or frequency were increased with NMN, sildenafil, amlodipine, TRAIL, and cytochalasin D. In old mice only, bosentan and DOI increased fenestration porosity and/or frequency. Modification of the actin cytoskeleton was observed with all agents that increased fenestrations, while NO synthase was only increased by sildenafil, amlodipine, and TRAIL. In conclusion, agents that target NO, actin, or lipid rafts promote changes in fenestrations in mice LSECs. Regulation of fenestrations occurs via both NO-dependent and independent pathways. This work indicates that age-related defenestration can be reversed pharmacologically, which has potential translational relevance for dyslipidemia and insulin resistance.

**NEW & NOTEWORTHY** We demonstrate the effects of multiple nitric oxide-dependent and -independent pharmaceutical agents on fenestrations of the liver sinusoidal endothelium. Fenestrations are reorganized in response to nicotinamide mononucleotide, sildenafil, amlodipine, and TNF-related apoptosis-inducing ligand. This work indicates that age-related defenestration can be reversed pharmacologically, which has potential translational relevance for dyslipidemia and insulin resistance in old age.

## INTRODUCTION

Liver sinusoidal endothelial cells (LSECs) have a unique morphology that promotes bidirectional exchange of substrates between the lumen of the hepatic sinusoid and the surrounding hepatocytes (9). This transfer function is thought to be predominantly performed by transcellular pores called fenestrations, located within the cytoplasmic extensions of LSECs. Fenestrations are 30–300 nm in diameter, and the majority of them are arranged in groups of 10–100 called liver sieve plates (9, 18). We have previously demonstrated that fenestrations act as conduits for lipoproteins (20), pharmacological agents (26), and insulin (27). In health, size and number of fenestrations are dynamic and responsive to various stimuli, such as fasting, alcohol, and various other chemicals (9), but it is also recognized that the overall size and number of fenestrations are reduced in chronic disease and aging (22). Loss of fenestrations in these and other experimental settings is mechanistically linked to impairment of the transfer of substrates and contributes to hyperlipidemia and insulin resistance (21, 27).

Age-related loss of fenestrations is called pseudocapillarization and represents a potential therapeutic target for age-related insulin resistance and hyperlipidemia. Previously, we and others have demonstrated that several compounds [7-ketocholesterol, 2,5-dimethoxy-4 iodoamphetamine (DOI), cytochalasin D, and vascular endothelial growth factor (VEGF)] promote increased fenestrations in young mice, with some promising results in older mice (8, 11, 40). The regulation of fenestrations is thought to be dependent mainly on the effects of nitric oxide (NO) via at VEGF-related pathway (40). This subsequently affects lipid raft organization, allowing fenestrations to form in the more fluid nonraft lipid membranes (13, 28). Therefore, drugs acting on these pathways are likely to influence fenestrations.

Because fenestrations are smaller than the limits of resolution of standard light microscopy, visualization of fenestrations in the past has relied primarily on electron microscopy. The recent development of super-resolution light microscopy (10, 40) now permits resolution of fenestrations using light-based modalities, such as structured illumination microscopy. Direct stochastic optical reconstruction microscopy (dSTORM) has also been applied to visualize LSEC fenestrations (28, 29). dSTORM is a super-resolution technique that uses photo-switchable fluorophores, single-molecule localization, temporal separation, and image reconstruction to achieve an optical resolution down to 20–30 nm (43). Conventional organic fluorophores act as photo switches in oxidized-reducing buffer solutions. The stochastic activation and temporal separation of the fluorophores/photo switches produce single-molecule “blinking” with nanometer localization precision. Up to 40,000 images are taken and reconstructed to produce a super-resolution image. With the use of plasma membrane stain DiD and actin stain, discrete cell membrane structures, such as sieve plates, lipid rafts, and fenestrations have be identified (28, 29). dSTORM, thus, provides an opportunity to study the effects of drugs on fenestrations using light microscopy and fluorophores.

Here, this exploratory study investigated the action of several agents on fenestrations in isolated LSECs from young (3–4 mo) and old (18–24 mo) mice to *1*) describe the different mechanisms that regulate fenestrations and *2*) identify drugs that reduce age-related loss of fenestrations. We studied drugs that act on the pathways that influence NO [sildenafil (2, 14), amlodipine (24, 46), and simvastatin (35, 38)], serotonergic pathway/phospholipase C (DOI) (31), endothelin receptor (bosentan) (34), death receptor (TNF-related apoptosis-inducing ligand, TRAIL) (7), and NAD^+^ (nicotinamide mononucleotide, NMN) (5) in mice using scanning electron microscopy (SEM) and dSTORM. Established fenestration-active agents (9) that act on the actin cytoskeleton and lipid rafts (cytochalasin D and 7-ketocholesterol, respectively) were used as positive controls. The results indicate that by targeting the NO pathway and inducing actin remodeling, we are able to promote refenestration in old mice. Agents that ameliorate age-related defenestration may have therapeutic potential for age-related dyslipidemia and insulin resistance.

## MATERIALS AND METHODS

Male C57/BL6 mice, 3–4 and 18–24 mo old, were obtained from the Animal Resource Centre in Perth, Western Australia. Animals were housed at the ANZAC Research Institute animal house on a 12-h:12-h light-dark cycle and provided with ad libitum access to food and water. Mice were not fasted before euthanasia by a single intraperitoneal injection with 100 mg/kg ketamine and 10 mg/kg xylazine in saline. The study was approved by the Animal Welfare Committee of the Sydney Local Health District and was performed in accordance with the Australian Code of Practice for the care and use of animals for scientific research (AWC 2016/009). All information provided accords with the ARRIVE (Animal Research: Reporting of In Vivo Experiments) guidelines.

Reagents included collagenase (type 1, Cat No. 47D17410A, ProSciTech, Kirwan, QLD, Australia), RPMI-1640 (Sigma-Aldrich, Castle Hill, NSW, Australia), Percoll (Sigma-Aldrich), cytochalasin D (Cat No: c8273; Sigma-Aldrich), TRAIL (Cat No. 375-TL-010; R&D Systems, Braeside, VIC, Australia), bosentan (Cat No. S4220; Selleckchem, Houston, TX), 7-ketocholesterol (Cat No. c2394; Sigma-Aldrich), 2, 5-dihydroxyl-4-isoamphetamine (Cat No. 13885; Cayman Chemicals, Redfern, Australia), simvastatin (Cat No. S6196; Sigma-Aldrich), sildenafil citrate (Cat No. PZ0003; Sigma-Aldrich), nicotinamide mononucleotide (gift from Dr. Lindsay Wu, University of New South Wales, Australia), amlodipine besylate (Cat No. A5605, Sigma-Aldrich), and vascular endothelial growth factor (VEGF; Cat No. V4512; Sigma-Aldrich). Stains included Alexa Fluor 488 phalloidin (Cat No. A12379; Thermo Fisher, North Ryde, NSW, Australia), phosphorylated-endothelial nitric oxide synthase (eNOS; cat. no. 9571; Cell Signaling Technology, Danvers, MA), endothelial nitric oxide synthase (eNOS) (Cat No. 610296; BD Biosciences, North Ryde, NSW, Australia) Alexa Fluor 488 goat anti-rabbit, Cy3 goat anti-mouse (Cat No. R-37116, A-11003; Thermo Fisher). Assays were performed using in vitro toxicology assay kit, 3-(4,5-dimethylthiazol-2-yl)-2,5-diphenyltetrazolium bromide (MTT)-based (Cat No. TOX1-1KT; Sigma-Aldrich), and cyclic GMP ELISA kit (Cat No. 581021; Cayman Chemicals).

As described previously (12), mouse LSEC isolation was performed by perfusion of the liver with collagenase. Nonparenchymal cells were removed by a two-step Percoll gradient and Kupffer cells were removed by selective adherence to plastic. LSECs (seeded at 0.5×10^6^ cells/cm^2^) were cultured (37°C, 5% CO_2_) in serum-free RPMI-1640 for 3.5 h before use.

Cells were treated with various agents for 30 min to determine effects on fenestrations. All agents were dissolved in serum-free RPMI media. All experiments were performed in triplicate for both young and old mice. Actin was disrupted with 0.5 µg/ml cytochalasin D, and lipid rafts were disrupted with 3.6 and 1.8 µg/ml 7-ketocholesterol; dosages were selected on the basis of our previous study (40). The NO pathway was promoted with sildenafil (0.6, 0.3, 0.15, 0.05, and 0.015 µg/ml) [dosages based on previous work by Burgess et al. (6)], amlodipine (40, 20, 10, 5, and 1 ng/ml) [dosages based on previous work by Stangier and Su (39)], and simvastatin (1 and 0.1 µg/ml) [dosages based on previous work by Ziviani et al. (48)]. The serotonergic/phospholipase C pathway was promoted with DOI [0.1 µg/ml) (11)], and endothelin receptors were inhibited by bosentan (550, 55, and 5.5 ng/ml) [dosages based on previous work by van Giersbergen et al. (44)]. Death receptor 4 was promoted with TRAIL (100, 10, 1, 0.1, and 0.01 ng/ml) (dosages based on previous work by Bernardi et al. (4)], and NAD^+^ was promoted with NMN [5,000, 50, 10, 1, and 0.1 µg/ml) [dosages based on previous work by Trammell et al. (42)].

SEM was performed as previously described (12, 40). LSECs were fixed in 2.5% glutaraldehyde in 0.1 M sodium cacodylate buffer, osmicated, dehydrated in graded ethanol, and hexamethyl disilazane, mounted on stubs, sputter coated with platinum, and examined using a JEOL 6380 Scanning Electron Microscope (JEOL, Tokyo, Japan). Images at ×10,000 magnification were collected by a blinded observer and used to measure fenestration diameter and LSEC porosity using ImageJ [National Institutes of Health (NIH), Bethesda, MD]. Between 616 and 3,312 fenestrations were counted per treatment. Fenestrations less than 30 nm and gaps more than 300 nm were excluded from analysis. Porosity was defined as the percentage of the cell membrane covered with fenestrations. Frequency was defined as the number of fenestrations per 1 µm^2^.

dSTORM imaging was performed using an in-house microscope (Mao H, Diekmann R, Liang H, Cogger VC, Le Couteur DG, Lockwood GP, Hunt NJ, Schüttpelz M, Huser TR, Chen VM, McCourt PAG., unpublished data). LSECs were prepared for dSTORM by washing twice with PBS and fixation with 4% paraformaldehyde for 30 min. Then LSECs were washed twice with PBS, permeabilized with Triton-X for 90 s, blocked with 5% BSA for 1 h, and stained with Alexa Fluor phalloidin 488 (1:40) for 20 min prior to imaging. Cells were washed using PBS with 0.1% Tween and placed in OxEA buffer (30) for dSTORM visualization and image capture. The dSTORM used 488- and 647-nm excitation from diode-pumped lasers (Coherent, Santa Clara, CA). Excitation was delivered via a 1.49 NA ×60 oil-immersion total internal reflection fluorescence objective (Olympus, Macquarie Park NSW, Australia). Fluorescence was captured on two separate sCMOS cameras (Imaging Development Systems, Obersulm, Germany). Data were collected for up to 40,000 images at around 75 frames/s. Five to eight whole cell images were collected by an observer blinded to the experiment for each treatment dosage and processed using rapidSTORM open source software (45). Each image was examined for all sieve plates and actin structures. Densitometry measurements were performed using 5–8 dSTORM images with data analysis performed using ImageJ software (NIH, Bethesda, MD).

Immunofluorescence was performed on LSECs fixed with 4% paraformaldehyde. LSECs were permeabilized with Triton-X for 90 s, blocked with 5% normal goat serum for 1 h and incubated with (1:100) phosphorylated-eNOS and (1:100) eNOS overnight at 4°C. LSECs were washed twice with PBS and incubated with Alexa Fluor anti-rabbit 488 and Alexa Fluor anti-mouse Cy3 secondary antibodies. Cells were washed with PBS and mounted using Vector Mount with DAPI. Slides were examined at ×63 magnification using a Leica SP8 inverted scanning confocal microscope with Type F immersion oil (cat. no. 11513859) and images captured using LAS software (Leica Microsystems CMS, Wetzlar, Germany) by a blinded observer. Images were analyzed using ImageJ (NIH).

Assays for MTT and cGMP were performed as instructed by the kit. Briefly, MTT assays were performed following drug treatments. Cells were washed with PBS and incubated with RPMI media containing 100 µg MTT solution. Cells were incubated at 37°C for 4 h and lysed with 200 µl of solubilization solution, and 30 min of color development followed and measured at 570 nm using a spectrophotometer. cGMP assays were also performed after drug treatments. Cells were washed with PBS and lysed with 0.1 M HCl. Following sample collection, the sample was acetylated and prepared with kit reagents. Samples were incubated for 18 h at 4°C before examination at 410 nm with a spectrophotometer.

Statistical analysis between drug treatments experiments and actin/NOS densitometry was performed comparing multiple groups using Kruskal-Wallis tests with a post hoc Dunn’s method (SPSS version 21; IBM Analytics, Melbourne, Australia) with *P* < 0.05 considered significant; *P* < 0.1 are also highlighted in the results. Nonparametric statistics were used due to the number of mice used in this study, with analysis of previous data demonstrating this sample size produces a statistical power of 80–95% to discriminate between interventions. Individual specifications of analyses are described in figures legends. All data are presented as means ± SD. Experimental design and analysis were performed in accordance with the American Physiological Society guidelines described in Curran-Everett and Benos (15).

## RESULTS

### 

#### Young and old controls.

**Fig. 1.**
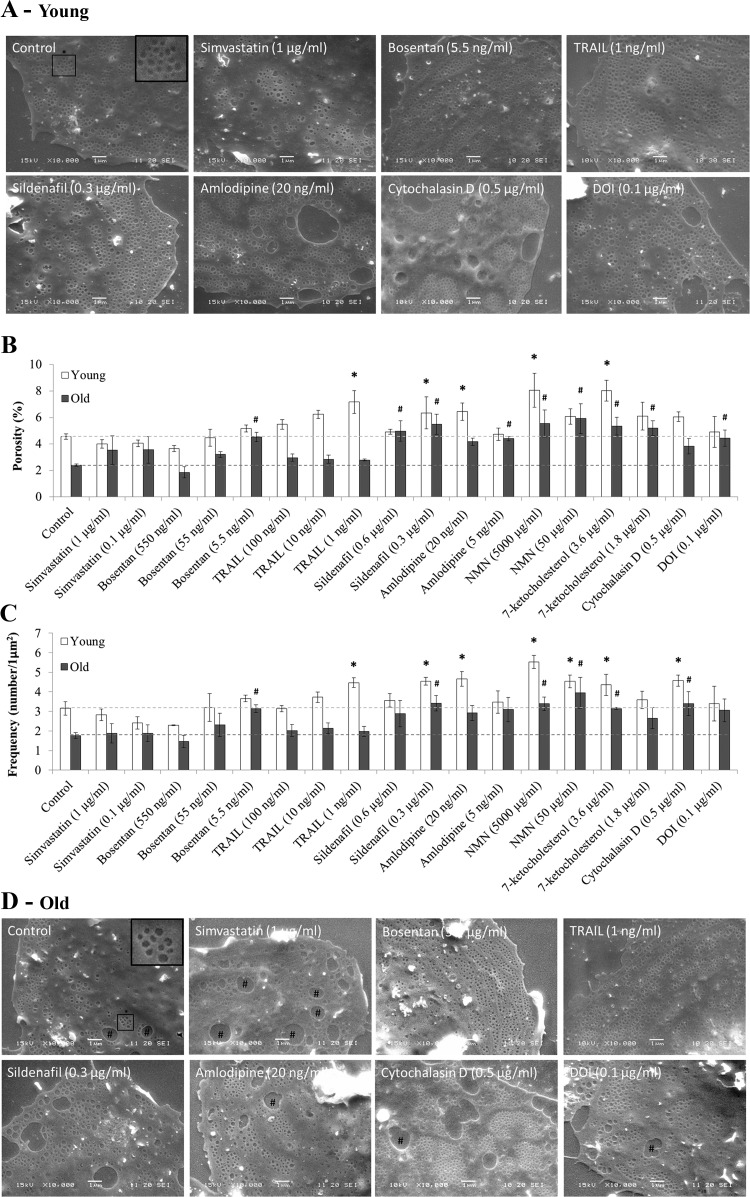
Effects of drug treatments on liver sinusoidal endothelial cells (LSEC) fenestration porosity and frequency in young and old mice. *A*: sample scanning electron microscopy (SEM) images of drug treatments in young mice. Control images show fenestrations grouped in sieve plates (see asterisk). Bosentan, TNF-related apoptosis-inducing ligand (TRAIL), amlodipine, sildenafil, and cytochalasin D treatments were maintained in sieve plates. Changes in fenestration %porosity (*B*) and frequency (number per 1 µm^2^) (*C*) following drug treatments in young (open bars) and old (gray bars) mice. Dashed lines show young and old control levels. The drug treatments used were simvastatin, bosentan, TRAIL, sildenafil, amlodipine, nicotinamide mononucleotide (NMN), 7-ketocholesterol, cytochalasin D, and 2, 5-dimethoxy-4-iodoamphetamine (DOI). All treatments were incubated at 37°C, 5% CO_2_ for 30 min using RPMI with or without dissolved drug. Scanning electron miscroscopy (SEM) images at ×10,000 were taken by two researchers blinded to the experiment (sample images *A* and *D*) and used to manually count fenestration porosity and frequency. Each data point represents the average ± SD of eight images, using 616–3,312 fenestration raw data points per treatment. All fenestrations <30 and gaps >300 nm were excluded from analysis. *P < 0.05 compared with young control; #*P* < 0.05 compared with old control. Statistics were performed using Kruskal-Wallis with post-hoc Dunn’s test to compare between groups; *n* = 3 for all groups. *D*: sample SEM images of drug treatments in old mice. All scale bars: 1 µm. Gaps (# signs) (>300 nm) were present in control and increased in simvastatin 1 µM treatments.

**Fig. 2.**
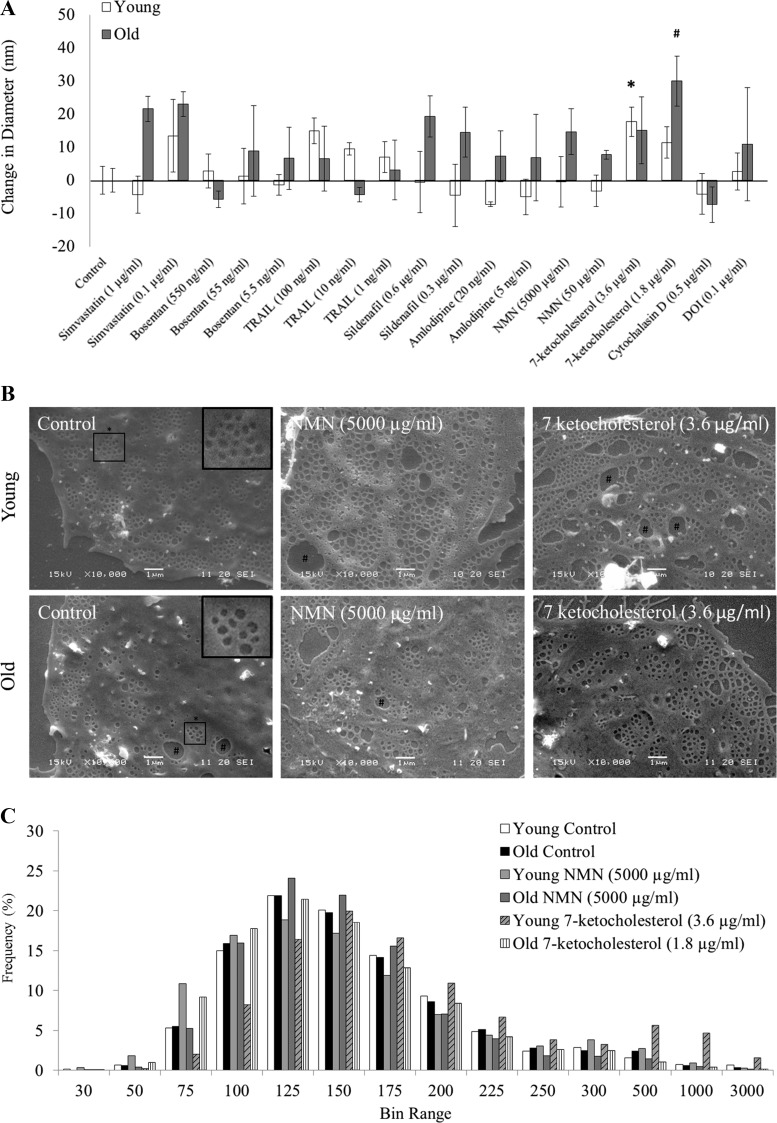
Effects of various drug treatments impacting liver sinusoidal endothelial cells (LSEC) fenestration diameter. *A*: changes in fenestration diameter induced by various drug treatments in young (open bars) and old (gray bars) mice. Drug treatments: simvastatin, bosentan, TRAIL, sildenafil, amlodipine, nicotinamide mononucleotide (NMN), 7-ketocholesterol, cytochalasin D, and 2, 5-dimethoxy-4-iodoamphetamine (DOI). All treatments were incubated at 37°C, 5% CO_2_ for 30 min using RPMI with or without dissolved drug. Manual counts of fenestration diameter were performed using scanning electron microscopy images at ×10,000. Data are presented relative to the %change compared with a control baseline. Each data point represents the average ± SD of eight images, using 616–3,312 fenestration raw data points per treatment. All fenestrations were <30 nm, and gaps >300 nm were excluded from analysis. *P < 0.05, compared with young control; #*P* < 0.05, compared with old control. Statistics were performed using Kruskal-Wallis with post hoc Dunn’s test to compare between groups; *n* = 3 for all groups. *B*: sample scanning electron microscopy (SEM) images of NMN and 7-ketocholesterol drug treatments in young and old mice. Scale bars of 1 µm are shown. Gaps (see #signs) (>300 nm) were present in 7-ketocholesterol treatments. NMN treatments maintained sieve plates, while 7-ketocholesterol treatments reduced lipid raft area. *C*: histogram of fenestration diameter in young control (white bars), old control (black bars), young NMN (light gray), old NMN (dark gray), young 7-ketocholesterol (hatched bars), and old 7-ketocholesterol (lined bars). Data are presented using the %frequency of diameters within the bin ranges shown.

SEM of isolated LSECs from young and old mice confirmed the technical success of LSEC preparations as shown in Fig. 1*A*. As expected, LSECs from old mice had reduced porosity when compared with young LSECs (porosity: young 4.6 ± 0.3 vs. old 2.4 ± 0.1%; *P* = 0.023; *n* = 3 per group, Fig. 1*B*), with a greater number of gaps (>300 nm in diameter, indicated by # in Fig. 1*D*). There was no significant difference in fenestration diameter with age (young 130.9 ± 7.2 vs. old: 124.4 ± 6.2 nm; *P* = 0.20, Fig. 2). There was a reduction in fenestration frequency with age (young: 3.1 ± 0.6 fenestrations per 1 µm^2^ vs. old 1.8 ± 0.3; *P* = 0.033, Fig. 1*C*). This indicates that age-related defenestration in these mice was largely secondary to reduced frequency of fenestrations rather than a reduction in diameter.

#### Effects of agents on fenestrations.

Treatment with sildenafil, NMN and 7-ketocholesterol led to significant increases in porosity and fenestration frequency in both young and old LSECs (Fig. 1, *B* and *C* and Tables 1 and 2). Cytochalasin D significantly increased frequency, but not porosity, in young and old LSECs (Fig. 1, *B* and *C* and Tables 1 and 2). LSECs from old mice only were responsive to bosentan and DOI. LSECs from young mice only demonstrated significant increases in porosity and frequency following TRAIL and amlodipine treatments. Overall, greater fold changes in porosity and frequency were observed in LSECs from old mice compared with young mice. The greatest changes on old mice were promoted by a 50 µg/ml NMN treatment, porosity increased by 2.5-fold and frequency by 2.25-fold (Fig. 1, *B*–*D*).

**Table 1.** Young mice data

Drug Treatment	Porosity, %	Diameter, nm	Frequency, No./Area
Control	4.55 ± 0.34	130.89 ± 7.23	3.15 ± 0.60
Amlodipine, 20 ng/ml	6.44 ± 1.14*	123.66 ± 1.18	4.67 ± 0.65*
Amlodipine, 5 ng/ml	4.73 ± 0.80	125.87 ± 9.30	3.47 ± 1.00
Bosentan, 550 ng/ml	3.66 ± 0.41	133.73 ± 9.03	2.30 ± 0.04
Bosentan, 55 ng/ml	4.48 ± 1.12	132.12 ± 9.03	3.20 ± 1.23
Bosentan, 5.5 ng/ml	5.18 ± 0.45	129.55 ± 5.46	3.66 ± 0.30
Cytochalasin D, 0.5 µg/ml	6.05 ± 0.62#	126.78 ± 10.68	4.57 ± 0.51*
DOI, 1 µg/ml	1.45 ± 1.27	135.64 ± 21.21	0.83 ± 0.75
DOI, 0.1 µg/ml	4.91 ± 2.02	133.57 ± 9.78	3.40 ± 1.56
NMN, 5,000 µg/ml	8.05 ± 2.23*	130.48 ± 13.21	5.53 ± 0.58*
NMN, 50 µg/ml	6.08 ± 1.00	127.68 ± 8.19	4.53 ± 0.58*
Sildenafil, 0.6 µg/ml	4.92 ± 0.35	130.33 ± 16.02	3.56 ± 0.61
Sildenafil, 0.3 µg/ml	6.34 ± 2.09*	126.34 ± 16.36	4.54 ± 0.34*
Simvastatin, 1 µg/ml	3.99 ± 0.55	126.55 ± 9.64	2.83 ± 0.51
Simvastatin, 0.1 µg/ml	4.05 ± 0.41	144.35 ± 18.95	2.42 ± 0.55
TRAIL, 100 ng/ml	5.49 ± 0.63	145.87 ± 6.77	3.15 ± 0.27
TRAIL, 10 ng/ml	6.24 ± 0.52#	140.36 ± 3.22	3.73 ± 0.46
TRAIL, 1 ng/ml	7.17 ± 1.49*	137.95 ± 8.12	4.47 ± 0.43*
7-ketocholesterol, 3.6 µg/ml	8.03 ± 1.37*	148.59 ± 7.70*	4.36 ± 0.93*
7-ketocholesterol, 1.8 µg/ml	6.10 ± 1.82	142.35 ± 8.02	3.59 ± 0.78

All data are shown as means ± SD. We used the Kruskal-Wallis test with post-hoc Dunn’s test to make between-group comparisons.

* *P* < 0.05;

# *P* < 0.1.

**Table 2.** Old mice data

Drug Treatment	Porosity, %	Diameter, nm	Frequency, No/Area
Control	2.40 ± 0.14	124.35 ± 6.15	1.77 ± 0.25
Amlodipine, 20 ng/ml	3.98 ± 0.48#	125.00 ± 13.36	3.00 ± 0.66
Amlodipine, 5 ng/ml	4.44 ± 0.29*	119.67 ± 22.63	3.56 ± 1.07#
Bosentan, 550 ng/ml	1.86 ± 0.72	118.64 ± 4.36	1.46 ± 0.54
Bosentan, 55 ng/ml	3.21 ± 0.36	121.14 ± 23.80	2.31 ± 1.03
Bosentan, 5.5 ng/ml	4.53 ± 0.59*	131.03 ± 16.29	3.14 ± 0.35*
Cytochalasin D, 0.5 µg/ml	3.82 ± 1.01	117.04 ± 9.26	3.39 ± 1.07*
DOI, 1 µg/ml	1.31 ± 0.47	155.28 ± 15.33*	0.67 ± 0.31
DOI, 0.1 µg/ml	4.44 ± 1.07*	135.27 ± 29.71	3.06 ± 1.00#
NMN, 5,000 µg/ml	5.55 ± 1.75*	139.05 ± 11.97	3.39 ± 0.60*
NMN, 50 µg/ml	5.92 ± 1.94*	132.12 ± 2.28	3.95 ± 1.35*
Sildenafil, 0.6 µg/ml	4.97 ± 1.34*	143.69 ± 10.80	2.88 ± 01.16
Sildenafil, 0.3 µg/ml	5.49 ± 1.33*	138.91 ± 13.05	3.41 ± 0.68*
Simvastatin, 1 µg/ml	3.54 ± 1.86	145.96 ± 6.55#	1.88 ± 0.86
Simvastatin, 0.1 µg/ml	3.56 ± 1.75	147.47 ± 6.47#	1.89 ± 0.74
TRAIL, 100 ng/ml	2.97 ± 0.46	130.88 ± 17.00	2.03 ± 0.53
TRAIL, 10 ng/ml	2.85 ± 0.53	120.08 ± 3.74	2.13 ± 0.48
TRAIL, 1 ng/ml	2.79 ± 0.14	127.50 ± 15.53	1.97 ± 0.49
7-ketocholesterol, 3.6 µg/ml	5.34 ± 1.17*	139.46 ± 17.45	3.15 ± 0.08*
7-ketocholesterol, 1.8 µg/ml	5.19 ± 0.95*	154.41 ± 13.07*	2.65 ± 0.94

All data are shown as means ± SD. We used Kruskal-Wallis with post-hoc Dunn’s test to make between-group comparisons.

* *P* < 0.05;

# *P* < 0.1.

There were significant differences in the responses of LSECs to different drug agents and dosages. In young mice, sildenafil (0.3 µg/ml), amlodipine (20 ng/ml), and TRAIL (1 ng/ml) demonstrated increased fenestration numbers and overall fenestrated cell area with some disruption to sieve plate formation (Fig. 1); higher dosages of sildenafil and TRAIL did not promote greater changes in fenestration porosity or frequency. Gap formation was apparent following treatment with amlodipine, 7-ketocholesterol, and NMN (indicated by # sign in Fig. 1*A* and Fig. 2*B*). Following NMN treatment, some normal sieve plates were maintained; however, there was a significant reduction of cytoplasmic area between sieve plates, resulting in a hyperfenestrated morphology, similar to the effects seen with 7-ketocholesterol in this study and reported previously (40). 7-ketocholesterol was associated with increased fenestration diameter in both young and old mice (*P* < 0.05; Fig. 2*A*).

There were effects of the drugs on the frequency distribution of fenestration diameter. NMN was associated with smaller fenestrations (less than 75 nm) on the edge of sieve plates (Fig. 2, *B* and *C*) in young mice but not old mice. In young mice, NMN (5,000 µg/ml) induced an increase in 30–100 and 226–500-nm fenestrations with a reduction in 126–200-nm fenestrations (Fig. 2*C*). In older mice, NMN treatment shifted the diameter of fenestrations from a peak of 76–100 to 101–125 nm and was associated with a reduction in smaller fenestrations (diameter 30–100 nm) (Fig. 2*C*). This effect was not observed with 7-ketocholesterol (3.6 µg/ml) treatment; instead, a shift to the right with decreased 30–125-nm-diameter fenestrations and an increase in 150–3,000 sized fenestrations occurred in young mice. In old mice, 7-ketocholesterol (3.6 µg/ml) demonstrated a peak at 101–125 nm with increased 150–300-nm fenestrations similarly to NMN.

**Fig. 3.**
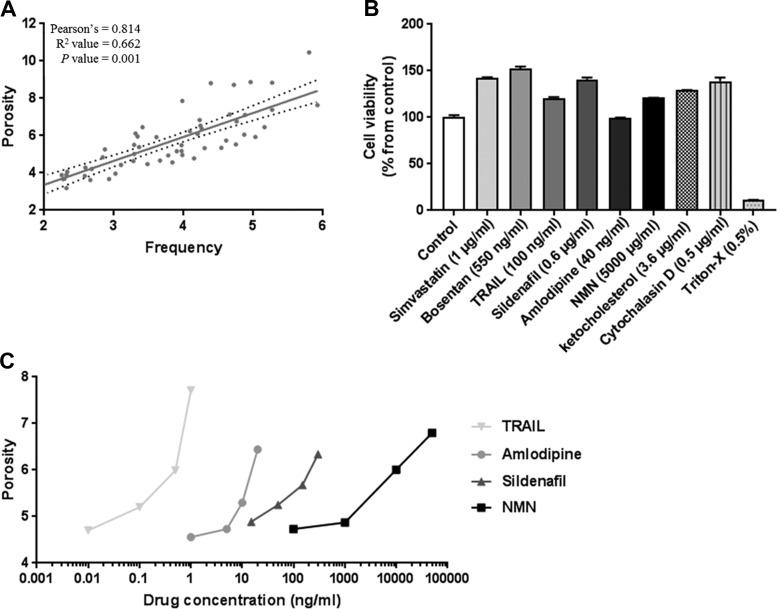
Correlations between porosity and frequency, cell viability, and dose-response curves relative to changes in porosity in young mice. *A*: correlation plot between %porosity and frequency in young and old mice. Data show all treatment data points (*n* = 3 for each group; 20 groups). *B*: cell viability at a percentage relative to controls. Sample data were collected in triplicate with error bars showing SD. *C*: dose-concentration curves relative to changes in %porosity of fenestrations. Data for young mice are shown; drug concentrations are shown as a log function.

Porosity was primarily increased as a result of increased numbers rather than the size of fenestrations (Fig. 3*A*). Cell viability was assessed via an MTT assay and demonstrated maximal drug dosages did not induce cellular toxicity (Fig. 3*B*). Dose-response experiments were performed in young mice for all drugs that were shown to be active in modulating fenestration porosity (Fig. 3*C*). TRAIL had the greatest activity and similar maximal efficacy to NMN but was more potent (Fig. 3*C*). Sildenafil, amlodipine, and TRAIL, however, had a limited dosage range for positive effects on fenestration porosity, while NMN had a broad range. NMN treatment resulted in the largest increase in fenestration porosity from 4.6 to 8.1% in LSECs from young mice.

#### Effects of agents on actin and nitric oxide synthase.

**Fig. 4.**
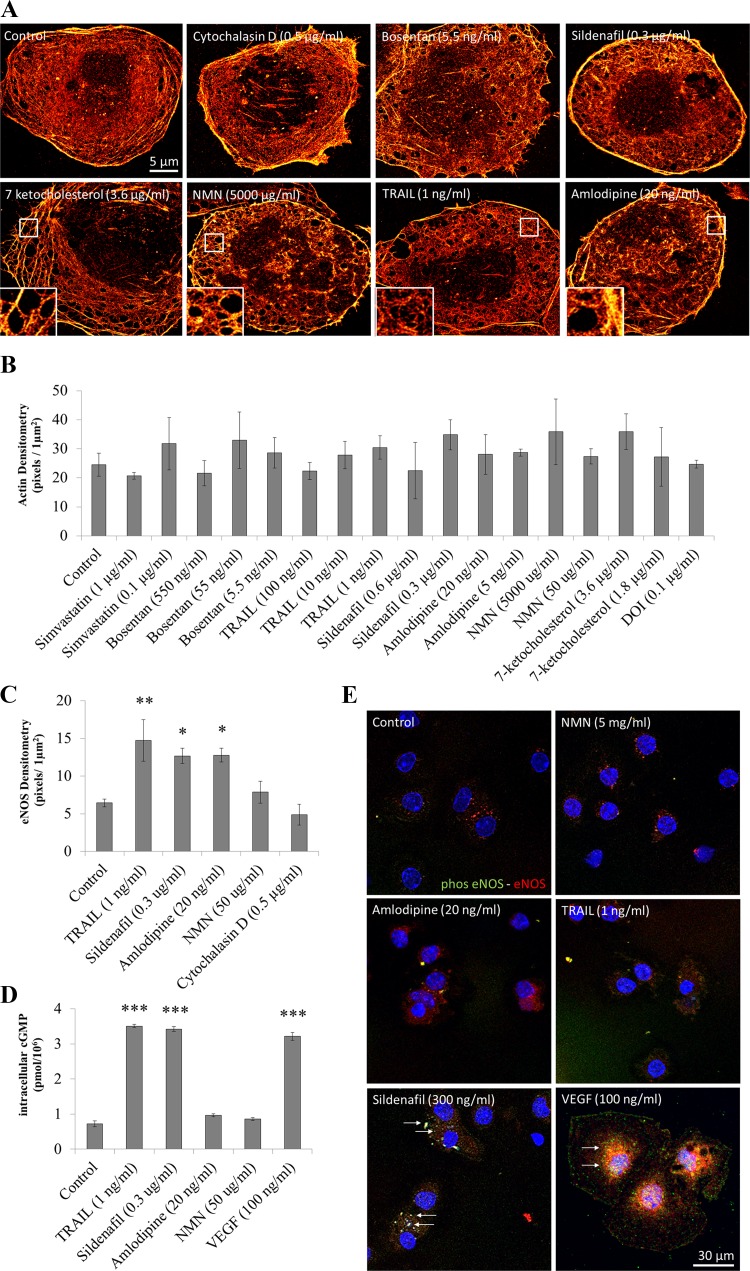
Effects of drug treatments on the actin cytoskeleton, nitric oxide synthase, and cyclic GMP. *A*: direct stochastic optical reconstruction microscopy (dSTORM) images showing actin cytoskeleton morphology changes promoted by various drug treatments in young mice. Images were produced following 40,000 image collections and processed using RapidSTORM software (45). Scale bar: 5 µm; insets show gaps and individual fenestrations within actin. *B*: changes in actin densitometry induced by drug treatments in young mice. Data are presented as a bar graph (means ± SD) with the density of pixels per 1 µm^2^. Eight images were captured using a dSTORM microscope (sample images shown in *A*); data analysis was performed using ImageJ software. Images were converted to binary data with measurements taken across the whole cell. *C*: changes in NOS densitometry induced by drug treatments in young mice. Data are presented as a bar graph (means ± SD) with the density of pixels per 1 µm^2^. Five images were captured using a dSTORM microscope; data analysis was performed using ImageJ software. Images were converted to binary data with measurements taken across the whole cell. *P < 0.05, **P < 0.01, and ***P < 0.01 using Kruskal-Wallis with post hoc Dunn’s test to compare between groups, data were duplicated in a second mouse to confirm observation. *D*: for intracellular cGMP, data are shown in pmol/10^6^, and error bars show SD. Assay was performed in triplicate with a biological replicate. ***P < 0.001 using Kruskal-Wallis with post-hoc Dunn’s test to compare between groups. *E*: immunofluorescent images of LSECs stained for phosphorylated NOS (green) and NOS (red). Scale bar: 30 µm. Control, NMN, and cytochalasin D demonstrated minimal staining, while sildenafil treatment promoted colocalization of phosphorated NOS and NOS (white arrows).

Control LSECs demonstrated moderate actin staining within the plasma membrane and cytoplasm, including broad circular tubular structures (Fig. 4*A*). No changes in actin density in LSECs were observed (Fig. 4*B*). Changes in the pattern of actin cytoarchitecture were observed between treatment groups (Table 3), while the overall quantity of actin in the cells was unchanged.

**Table 3.** Actin and nitric oxide synthase changes with drug treatments

Drug	Actin and NOS Changes
Cytochalasin D	Disordered actin structure Dense actin plasma membrane Stress fibers Single isolated fenestrations No NOS or pNOS changes
Amlodipine and NMN	Disordered actin structure Dense actin plasma membrane Actin clusters Gap formations Individual fenestrations Increased NOS in amlodipine
Sildenafil	Disordered actin structure Dense actin plasma membrane Gap formations Individual fenestrations Increased NOS and pNOS
TRAIL	Disordered actin structure Gap formations Minimal actin clustering Increased NOS
Bosentan	Stress fibers Fused actin structure Individual fenestrations
7-ketocholesterol	Ordered actin structure Extensive gap formations within the cytoplasm

LSECs treated with cytochalasin D had extensive actin staining of the plasma membrane (Fig. 4*A*). Stress fibers were present within the perinuclear area. There was a loss of smooth fibers encircling the cytoplasm following treatment with cytochalasin D, amlodipine, NMN, and sildenafil.

Sildenafil, amlodipine, and NMN demonstrated a similar phenotype with disordered, dense actin staining in the plasma membrane and clustering of actin within the cytoplasm (Fig. 4*A*). The key features were *1*) fibers projected in all directions, *2*) the presence of actin clusters, *3*) gap formation, and *4*) individual fenestrations visible in some sieve plates (Fig. 4*A*, *insets*). TRAIL was similar to sildenafil, amlodipine, and NMN apart from the absence of the intense actin clustering (Table 3 and Fig. 4*A*).

7-Ketocholesterol treatment was associated with organized actin structure throughout the cytoplasm similar to controls (Fig. 4*A*). However, large gaps occurred throughout the actin cytoarchitecture, actin fibers maintained their continuous and interconnected appearance but lost their circular tubular structures. Moderate staining was seen in the plasma membrane. The large gaps were also observed in the cytoplasmic actin (Fig. 4*A*, *inset*).

Changes in the actin cytoskeleton were associated with increased fenestration porosity and frequency; however, there did not appear to be any specific pattern of change in the cytoskeleton that was associated with increased fenestrations with all treatments.

Increased NOS densitometry was observed for TRAIL, amlodipine, and sildenafil (Fig. 4*C*). Intracellular cGMP was increased three-fold following sildenafil and TRAIL treatments (*P* = 0.001); no changes were observed in NMN or amlodipine-treated cells (Fig. 4*D*). Control LSECs and those treated with NMN demonstrated minimal NOS staining and nonphosphorylated NOS (Fig. 4*E*). TRAIL and amlodipine showed NOS staining across the cytoplasm but without phosphorylated NOS (Fig. 4*E*). Sildenafil and VEGF (100 ng/ml, 4 h treatment) showed staining for both NOS and phosphorylated NOS (Fig. 4*E*, white arrows).

## DISCUSSION

The morphology of fenestrations in LSECs is responsive to a variety of pharmacological interventions, and this responsiveness is mostly maintained into older age. LSECs isolated from old mice in this study had reduced porosity and frequency of fenestrations, consistent with previous studies in mice as well as rats, humans, and nonhuman primates (9, 23, 25). NMN, sildenafil, and 7-ketocholesterol increased fenestration porosity and frequency in young mice, with similar or greater effects seen in LSECs from old mice (summary data provided in Table 4). This indicates that age-related defenestration can be reversed in vitro and may be a valid therapeutic target for in vivo studies. Moreover, the optimal concentrations of these refenestrating agents were identified in LSECs from old mice, providing a potential target dose for in vivo studies. The results of the dSTORM studies showed that refenestration was associated with significant actin reorganization. Increased NOS protein expression was also seen in LSECs treated with amlodipine, sildenafil, and TRAIL, while sildenafil was the only agent associated with increased phosphorylation of NOS. Overall, our study indicates that agents that increased fenestrations are associated with an alteration of the actin cytoskeleton and in some cases, release of NO; importantly, this responsiveness is maintained in old age.

**Table 4.** Changes in fenestration porosity, diameter, and frequency promoted by various drugs and agents

Drug	Porosity		Diameter		Frequency	
	Young	Old	Young	Old	Young	Old
Simvastatin, 1 µg/ml				↑ NS		
Simvastatin, 0.1 µg/ml				↑ NS		
Bosentan, 550 ng/ml						
Bosentan, 55 ng/ml						
Bosentan, 5.5 ng/ml		↑				↑
TRAIL, 100 ng/ml						
TRAIL, 10 ng/ml	↑ NS					
TRAIL, 1 ng/ml	↑				↑	–
Sildenafil, 0.6 µg/ml	–	↑				
Sildenafil, 0.3 µg/ml	↑	↑			↑	↑
Amlodipine, 20 ng/ml	↑	↑ NS			↑	↑ NS
Amlodipine, 5 ng/ml		↑				
NMN, 5,000 µg/ml	↑	↑			↑	↑
NMN, 50 µg/ml		↑			↑	↑
7-ketocholesterol, 3.6 µg/ml	↑	↑	↑		↑	↑
7-ketocholesterol, 1.8 µg/ml		↑		↑		
DOI, 1 µg/ml				↑		
DOI, 0.1 µg/ml		↑				↑ NS
Cytochalasin D, 0.5 µg/ml	↑ NS	–			↑	↑

↑Increased (*P* < 0.05); NS, nonsignificant (*P* < 0.1).

In old mice, NMN (50 µg/ml) generated the greatest increase in fenestration porosity and frequency. NMN is a biosynthetic nicotinamide adenine dinucleotide (NAD^+^) metabolite that is critical for the regulation of NAD^+^ biosynthesis via the NAD^+^ salvage pathway (5, 33). NMN is converted to NAD^+^ by NMN acetyltransferase and is produced from the NAD^+^ breakdown product nicotinamide in the presence of nicotinamide phosphoribosyltransferase. This salvage process occurs in the nucleus, mitochondria, and cytosol and maintains high levels of NAD^+^ in the liver (47). Elevated NAD^+^ is promoted 15 min following a single intraperitoneal injection of 500 mg/kg NMN in female mice. In old rats, it has been shown that this dosage is nontoxic and promotes improved glucose tolerance (32). Similar dosages given continuously for 7 days were also shown to improve insulin action and secretion in diet- and age-induced Type 2 diabetic mice models (47). Our data suggest that one mechanism for the effects of NMN on glucose and insulin metabolism might involve refenestration of the old LSEC, leading to increased insulin sensitivity in the liver (27). In young LSECs, NMN (5,000 µg/ml concentration) generated increased fenestration porosity and frequency with shifts in the distribution of diameter. The fenestration diameter histogram (Fig. 2*C*) highlights the presence of small 30–100-nm fenestrations and larger 125–300 fenestrations following 30 min of treatment. NMN increased the frequency of fenestrations substantially, which suggests that the increase in the proportion of small fenestrations might represent the formation of new fenestrations. In old mice, NMN treatment shifted the diameter of fenestrations to the right, with an increase in fenestration diameter. Consequently, the average fenestration diameter in old mice treated with NMN was similar to young control mice (old NMN: 132 ± 2 vs. young control: 131 ± 7 nm).

These agents also had varying effects on the actin cytoskeleton as visualized using dSTORM. The condensation and clustering of actin appeared to be similar following treatment with cytochalasin D, amlodipine, sildenafil, and NMN. However, treatment with 7-ketocholesterol produced a diffuse and stretched actin network, possibly generated by the retraction of lipid rafts that are anchored to the actin cytoskeleton (13, 27), and this was associated with a significant 15-nm increase in fenestration diameter. This suggests that agents that act upstream on the actin cytoskeleton will largely influence frequency of fenestrations and agents that act directly on lipid rafts may additionally increase the diameter of fenestrations, perhaps as a result of increased nonlipid raft cell membrane.

The regulation of LSEC fenestrations has been recently reviewed, and the major regulatory pathway is thought to be mediated by VEGF and NO (16). We investigated three drugs that influence NOS and NO: amlodipine, sildenafil, and simvastatin. Only sildenafil influenced LSECs in both young and old mice, and amlodipine showed a similar pattern in fenestration changes but did not demonstrate statistical significance. Sildenafil promotes cGMP and PKG via inhibition of PKE5 leading to increase NO availability (14, 37). Amlodipine has a dual action on NO via cGMP and inhibition of Ca^2+^ channels (3, 24). Sildenafil does not inhibit Ca^2+^ influx (41). Simvastatin promotes the releases of NO from the endothelium via an Akt-dependent pathway (1) and inhibits Rho GTP-kinase to indirectly promote cGMP and PKG activation (19). Simvastatin does not promote Ca^2+^ flux (36). This study showed that sildenafil, and, to a lesser extent, amlodipine, promoted changes in fenestration porosity and frequency, with increased NOS expression. Simvastatin, in comparison, promoted a nonsignificant increase in fenestration diameter. These findings support the NO-cGMP-PKG pathway proposed but suggest that direct targeting of cGMP and PKG signaling (such as by sildenafil and amlodipine) may promote greater fenestration porosity and frequency and targeting Akt-dependent NO release via simvastatin may increase fenestration diameter. Future studies are required to determine whether these drugs increase fenestrations in old animals in vivo and whether this leads to increased hepatic clearance of circulating insulin and lipoproteins.

We also studied the effects of TRAIL. TRAIL is a death receptor agonist and promotes caspase-8-dependent programed cell death. In old mice, TRAIL had minimal effects on the LSEC, however, in young mice; TRAIL was associated with a 60% increase in porosity and a 40% increase in fenestration frequency. TRAIL had similar effects as sildenafil in terms of effects on fenestration frequency and diameter, actin, and NOS. TRAIL has been reported to upregulate NOS and phosphorylated NOS following 15 min of 1 µg/ml treatment in human umbilical vein endothelial cells (17). Together, these results indicate that among its other established effects, TRAIL also influences NOS expression in endothelial cells (7).

Previously, it has been reported that cytochalasin D, 7-ketocholesterol, and DOI increase fenestration porosity in young mice (11, 40) without any significant effects on fenestration diameter. In the recent studies, we observed increased porosity with 7-ketocholesterol only; however, cytochalasin D demonstrated a 33% increase but was not significant (*P* = 0.08). We also found that cytochalasin D, 7-ketocholesterol, but not DOI, increased fenestrations in LSECs from old mice. However, we previously reported that in vivo administration of DOI increased fenestrations only in young (7 mo) but not old (24 mo) mice (11). The difference in these results presumably reflects the different methodologies (in vivo vs. in vitro) and ages (18 mo vs. 24 mo) used in these studies.

In conclusion, we have shown that in vitro drug treatments with NMN, sildenafil, and 7-ketocholesterol increase fenestration porosity and frequency in LSECs isolated from young and old mice. The regulation of fenestrations may be mediated by NO-dependent and -independent pathways. Defenestration associated with age-related pseudocapillarization can be reversed by several different agents, which may have an impact on age-related dyslipidemia and hepatic insulin resistance.

## GRANTS

The study was supported by Australian National Health and Medical Research Council Project no. 1141234 and the Aging and Alzheimer’s Research Foundation (a Division of the Medical Foundation of the University of Sydney).

## DISCLOSURES

No conflicts of interest, financial or otherwise, are declared by the authors.

## AUTHOR CONTRIBUTIONS

N.J.H., G.P.L., A.W., H.M., and P.A.G.M. performed experiments; N.J.H., G.P.L., and A.W. analyzed data; N.J.H., D.G.L.C., and V.C.C. interpreted results of experiments; N.J.H. prepared figures; N.J.H. drafted manuscript; N.J.H., D.G.L.C., and V.C.C. edited and revised manuscript; N.J.H., D.G.L.C., and V.C.C. approved final version of manuscript.
